# Cogels of Hyaluronic Acid and Acellular Matrix for Cultivation of Adipose-Derived Stem Cells: Potential Application for Vocal Fold Tissue Engineering

**DOI:** 10.1155/2016/6584054

**Published:** 2016-11-17

**Authors:** Dongyan Huang, Rongguang Wang, Shiming Yang

**Affiliations:** Department of Otolaryngology-Head and Neck Surgery, Clinical Division of Surgery, Chinese PLA General Hospital, Beijing 100853, China

## Abstract

Stem cells based tissue engineering has been one of the potential promising therapies in the research on the repair of tissue diseases including the vocal fold. Decellularized extracellular matrix (DCM) as a promising scaffold has be used widely in tissue engineering; however, it remained to be an important issue in vocal fold regeneration. Here, we applied the hydrogels (hyaluronic acid [HA], HA-collagen [HA-Col], and HA-DCM) to determine the effects of hydrogel on the growth and differentiation of human adipose-derived stem cells (hADSCs) into superficial lamina propria fibroblasts. hADSCs were isolated and characterized by fluorescence-activated cell sorting. The results indicated that HA-DCM hydrogel enhanced cell proliferation and prolonged cell morphology significantly compared to HA and HA-Col hydrogel. Importantly, the differentiation of hADSCs into fibroblasts was also promoted by cogels of HA-Col and HA-DCM significantly. The differentiation of hADSCs towards superficial lamina propria fibroblasts was accelerated by the secretion of HGF, IL-8, and VEGF, the decorin and elastin expression, and the synthesis of chondroitin sulfate significantly. Therefore, the cogel of HA-DCM hydrogel was shown to be outstanding in apparent stimulation of hADSCs proliferation and differentiation to vocal fold fibroblasts through secretion of important growth factors and synthesis of extracellular matrix.

## 1. Introduction

Injury to the vocal fold can lead to intractable changes in the composition and distribution of extracellular matrix (ECM) in the lamina propria and also cause irreversible vocal scarring through significant fibrosis and deposition of hyperplasia disorderly [1–3]. With the development of tissue engineering, biomaterials and stem cells based regenerative therapy had been shown to be one of the promising alternative methods for the treatment of vocal fold injury [4, 5].

Cells and biomaterials are two indispensable elements in tissue engineering aiming at the construction of complex functional tissues* in vitro*. In previous studies, many different types of cells were used in tissue engineering [6–8]. Adipose-derived stem cells (ADSCs) as a population of pluripotent mesenchymal stem cells were promising in the application of regenerative medicine due to their capability of differentiation towards various lineages. A number of* in vivo* applications of ADSCs have been reported in diverse tissue and organ diseases [9–11]. Xu et al. stated that ADSCs showed a satisfactory effect on regeneration of vocal fold [12]. The injection of ADSCs derived fibroblast-like cells in the injured vocal fold was proved to heal the vocal fold wound through deposition of ECM compositions* in vivo* [13].

In tissue engineering, various hydrogels were applied as scaffolds for ADSCs with great potential, such as fibrin, collagen, and hyaluronic acid (HA) [14–16]. The outstanding scaffolds for tissue engineering should serve the following important duties: (1) supporting cell growth and differentiation of stem cells and (2) restoring the volume of the superficial lamina propria [17]. However, there are still some limitations in the application of only one element from the extracellular matrix (ECM) for cell culture such as the absence of essential growth factors for regulation of cell growth and differentiation of stem cells. In recent years, the application of composite materials in the culture of ADSCs has attracted great interest from researchers to induce differentiation into targeted cell lineages.

ECMs in vocal fold tissues are composed of an organized porous structure of various matrix macromolecules, mainly proteoglycans and glycosaminoglycans (GAGs), the fibrous proteins that are secreted by vocal fold fibroblasts. Collagen and elastin organized structural components of ECMs [18], while fibronectin and laminin formed the main adhesive elements of ECMs. Besides, in vocal fold lamina propria, hyaluronic acid, as another important ECM constituent involved in the regulation of tissue viscosity, osmosis, and dampening [19], and decorin, distributed throughout the vocal fold lamina propria, enhance the assembly of collagen fibers and bundles.

HA as an abundant component of normal vocal fold was shown to be promising in vocal fold tissue engineering. The scaffolds composed of HA provided physiochemical cues for cell growth and differentiation [17, 20]. Collagen as another major component of ECM was applied in tissue engineering widely, and also the composite biomaterials of collagen and other materials including HA showed unique advantages for cell differentiation [21]. However, collagen hydrogel composed of collagen was still limited to some cytokines and some functional proteins for cell growth and differentiation. Recently, decellularized extracellular matrix (DCM) was shown to be promising in tissue engineering due to its natural derivation and functional factors for cell growth [22–24]. However, there are still few studies about its application in vocal fold regeneration; in order to evaluate the application of DCM in vocal fold tissue engineering, we used cogel of HA-DCM to construct hydrogel-MSCs constructs to understand the growth and differentiation of hADSCs in the 3D environment.

Here, we used cogel of HA-DCM and hADSCs to construct 3D hydrogel-cell constructs and evaluate the effect of HA-DCM hydrogel on the growth and differentiation of hADSCs. Meanwhile, through analyzing the secretion of cytokines and synthesis of ECM, we will figure out how HA-DCM hydrogel affects the differentiation of hADSCs towards vocal fold fibroblasts. This study aims to find a promising cogel to stimulate the differentiation of stem cells towards vocal fold fibroblasts and to be used for regeneration of injured vocal fold.

## 2. Materials and Methods

### 2.1. Isolation of hADSCs

hADSCs were isolated from human adipose tissues by digestion of collagenase and filtration using cell strainers as in previous methods [17]. Human abdominoplasty adipose tissues were obtained from 20- to 30-year-old female donors (*n* = 5), and the collected specimens were washed with saline buffer five times to remove blood and free fatty acids. The washed adipose tissues were cut into small blocks and then were digested with Blendzyme (0.3 mg/mL) for 30 min while stirring at 37°C. The incubated tissues were filtered through 100 and 40 *μ*m cell strainers to remove fibrous redundant tissues and then centrifuged at 700 ×g for 10 min. The cells were collected to remove red blood cells using a lysis buffer and then centrifuged again as the previous condition. The collected cells were resuspended in *α*-MEM and cultured in 37°C, 5% CO_2_ humid incubator.

### 2.2. Characterization of hADSCs

hADSCs were characterized by fluorescence-activated cell sorting (FACS) analysis. In brief, hADSCs were incubated with fluorescent primary antibodies including CD105, CD90, CD34, and CD45 for 30 min at room temperature. The incubated cells were washed by phosphate buffered saline (PBS) buffer containing 5% serum (v/v) three times. The labeled cells were analyzed using a BD FACSCalibur™ system.

### 2.3. Transduction of hADSCs with Green Fluorescence Protein (GFP)

In order to visualize hADSCs in 3D cell culture conditions, the hADSCs were transfected with lentivirus with expression of GFP as in previous methods [17]. Lentivirus expressing GFP was expanded by 293T cells. 500,000 hADSCs were transfected with GFP-lentivirus for 6 h, and the infected efficiency of GFP-lentivirus was examined by GFP expression using FACS (BD Company). The transfected hADSCs showed no apparent difference in the morphology with untreated hADSCs in 2D culture.

### 2.4. Construction of 3D Constructs of Cells Embedded in Hydrogel

Small intestinal submucosa derived DCM was purchased from Cook Biotech (West Lafayette, IN, USA). HA (Restylane) hydrogel with the concentration 20 mg/mL was formed as the manufacturer's protocol. Collagen type I gel was purchased from BD. DCM solution was prepared by dissolving 1 g DCM powder and 50 mg of pepsin in 0.01 M HCl while stirring for 48 h at room temperature. The solution of DCM was diluted with PBS to 3 mg/mL, and also the pH was modulated to 7.4 with 0.1 N NaOH. Composite hydrogels of HA-collagen and HA-DCM were produced by mixing the composites of HA and collagen or DCM at the ratios of 1 : 1. The HA-Col composite hydrogel was cross-linked as in the provided protocol. 3D cell-hydrogel constructs were obtained by mixing hADSCs (200,000 cells) at passage 3 in 200 *μ*L hydrogels and were cultured for 7 days in H-DMEM with supplementation of 5% FBS in a 37°C, 5% CO_2_ incubator. The culture media were replenished every 2 days.

### 2.5. The Secretion of HGF, IL-8, VEGF, and Chondroitin Sulfate in hADSCs by Analysis of ELISA

ELISA was applied to determine the secretion of HGF, IL-8, and VEGF of hADSCs on the surface of HA, HA-Col, and HA-DCM hydrogel. HA, HA-Col, and HA-DCM hydrogels were coated in 12-well plates, and hADSCs (50,000 cells) at passage 3 were seeded on the surface of HA, HA-Col, and HA-DCM hydrogel individually. Conditioned media from three groups were collected at 72 hours as in previous methods. Human HGF, IL-8, VEGF, and chondroitin sulfate ELISA kits from R&D Systems were used as per the manufacturer's description to analyze the concentration of HGF, IL-8, VEGF, and chondroitin sulfate in the media. All ELISA experiments were repeated three times.

### 2.6. Proliferation of hADSCs in Hydrogel by DNA Content

In order to determine the proliferation of hADSCs in hydrogel, 3D cell-hydrogel constructs was collected to measure the total DNA content. The PicoGreen kit (Molecular Probes, Eugene, OR) was used as per the manufacturer's instruction. In brief, the extraction buffer containing 1 N NH_4_OH and 0.2% Triton X-100 was used to suspend 3D constructs of cell-hydrogel, and a bead beater from BioSpec was used to release DNA. The extract was analyzed using PicoGreen following the manufacturer's instruction after removal of debris through centrifugation at 10,000 ×g for 10 min.

### 2.7. Immunofluorescence Staining

Immunofluorescence staining of 3D cell-hydrogel constructs was conducted as in a previous method. In brief, the constructs were collected, fixed by 4% paraformaldehyde, and paraffinized. Sections were deparaffinized and antigen was treated by heat for 20 min at 95°C. The sections were blocked with horse serum for 30 min at room temperature and incubated with CD105 primary antibody (Abcam, Cambridge, MA) in a 4°C refrigerator overnight. FITC-conjugated secondary antibody (Abcam, Cambridge, MA) was incubated at room temperature for 2 h in the dark. Nuclei were stained by DAPI for 20 min in the dark. The pictures were observed by a laser microscope (Leica).

### 2.8. Real-Time-Polymerase Chain Reaction (RT-PCR)

The important constituents of ECMs, decorin and elastin, were determined by RT-PCR [17]. Total RNA of cell-hydrogel constructs was prepared using an RNeasy system (Qiagen, Valencia, CA) and a bead beater (BioSpec Products, Bartlesville, OK). The extracted total RNA was treated with DNase for 20 min to remove genomic DNA from the samples. Then, cDNA was synthesized using oligo-dT primers from the total mRNA (Invitrogen), and 200 ng of the cDNA was used for the real-time PCR analysis (AB7500). TaqMan Universal PCR mix, gene-specific primers, and cDNA were mixed, and the PCR reaction was performed at 50°C for 2 min, 95°C for 10 min, and 40 cycles of 95°C for 15 s and 60°C for 1 min. GAPDH was used as the control. The expression of gene level, decorin and elastin, was calculated as the following equation: relative expression = 2/(Ct_sample_ − Ct_GAPDH_), where Ct indicates the average cycle threshold (Ct) of three replicates. Three samples from each group were used for gene analysis.

### 2.9. Statistical Analysis

Average and standard deviations were obtained from three separate experiments. All data are expressed as mean ± standard error of the mean (SEM) unless indicated otherwise and analyzed using one-way analysis of variance (ANOVA) using Tukey's* post hoc* test. Data was considered statistically significant at *p* < 0.05.

## 3. Results

### 3.1. Isolation, Culture, and Characterization of hADSCs

**Figure 1:**
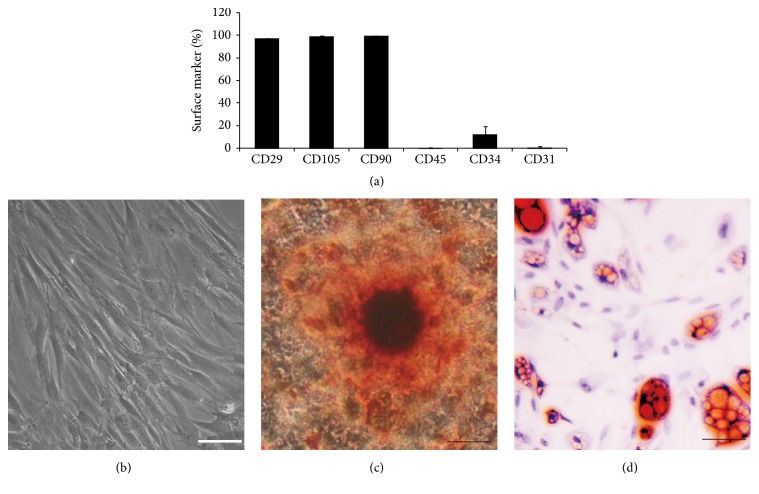
Characterization of isolated hADSCs. (a) Isolated hADSCs were shown to be positive for CD29 (98.9%), CD105 (99.4%), and CD90 (99.7%), while they have been shown to be negative for CD45 (0.1%), CD34 (16.4%), and CD31 (0.1%) in cell population. (b) hADSCs were shown to be in spindle-like shape at passage 3. Scale bar = 50 *μ*m. (c) Osteogenesis of hADSCs was assessed by von Kossa method. (d) Adipogenesis of hADSCs was assessed by Oil Red-O staining.

Adipose tissue is composed of various types of cells such as endothelial cells, adipose cells, fibroblasts, and adipocyte progenitors. We isolated adipose-derived stem cells using a mixture of collagenase and dispase as in previous methods [17]. Fresh isolated cells were cultured in cell culture dishes at a density of 35,000 cells/mm^2^. In order to characterize hADSCs, expression of surface markers in cells was analyzed by FACS after 3 passages as in previous methods [25, 26] (Figure 1(a)). The results showed that more than 90% of cells expressed CD105 (99.4%) and CD90 (99.7%), and most of the cells were shown to be negative for CD34 (16.4%) and CD45 (0.1%) which were the surface markers of hematopoetic stem cells. Meanwhile, cells were shown to be in spindle shape at day 4 of culture (Figure 1(b)).

To further characterize the multipotentiality of hADSCs, the osteogenic and adipogenic differentiation of hADSCs was determined. hADSCs were cultured in the osteogenic media and were positively stained with von Kossa method, which indicated the existence of osteoblasts (Figure 1(c)), while hADSCs showed positive staining with Oil Red-O, indicative of adipocytes (Figure 1(d)). Based on the FACS data and differentiation results, we concluded that the cell populations are enriched with stem cells, which were referred to as hADSCs.

### 3.2. Cell Morphology of hADSCs in HA-DCM Composite Hydrogel

**Figure 2:**
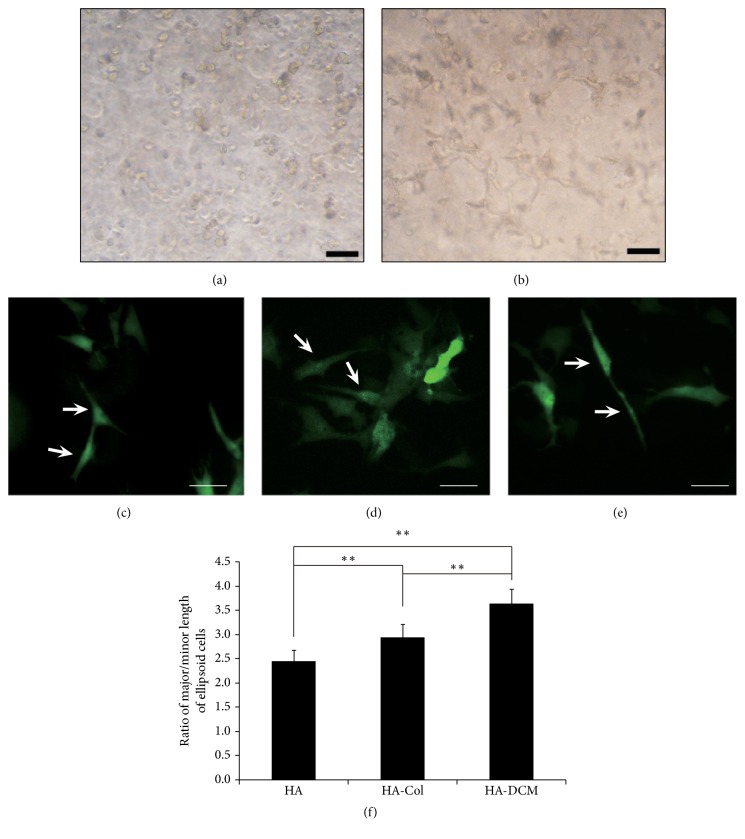
Cell morphology of hADSCs in 3D hydrogel was determined in the cell culture. (a) hADSCs were shown to be circular at 0 h in HA-DCM composite hydrogel; (b) hADSCs developed into spindle cells with fibroblasts-like shape at 24 h; (c–e) cell shape of hADSCs that were labeled with GFP in HA, HA-Col, and HA-DCM hydrogel was observed at the 7th day after cell seeding. The arrows indicated that hADSCs in HA, HA-Col, and HA-DCM hydrogel were shown to be ellipsoid at the 7th day. (f) Quantification of cell shape of hADSCs indicated that the major/minor length of hADSCs in HA-DCM hydrogel was shown to be larger than one in HA-Col hydrogel, and the major/minor length of hADSCs in HA hydrogel was shown to be the smallest in the three groups. *∗∗* indicated *p* < 0.05.

Cell shape affected the cell proliferation and differentiation of hADSCs towards different lineages. In order to understand the effects of HA-DCM composite hydrogel on the cell morphology of hADSCs, we transfected hADSCs by GFP-lentivirus and visualized the GFP positive cells using light microscope and fluorescence microscope as in a previous method [17] (Figure 2). hADSCs were shown to be round when they were embedded in composite hydrogel on the first day (Figure 2(a)), and hADSCs elongated significantly within HA-DCM composite hydrogel in a time-course manner (Figure 2(b)). Through visualization of GFP, cells exhibited obvious variance in cell shape significantly (Figures 2(c)–2(e)). Some of the hADSCs were shown to have fibroblast-like shape in HA-DCM hydrogel, but apparently more hADSCs with spindle or ellipsoid shape existed in both HA-Col and HA-DCM hydrogel than in HA hydrogel. Furthermore, the ratio of major/minor length through quantification of cell shape by ImagJ2x software indicated that HA-DCM hydrogel induced the change of cell shape of hADSCs with greater ratio of major/minor length of ellipsoid cells significantly compared to HA-Col and HA hydrogel (Figure 2(f)).

### 3.3. Cell Growth and Differentiation of hADSCs in Hyaluronic Acid/Acellular Matrix Composite Hydrogel

**Figure 3:**
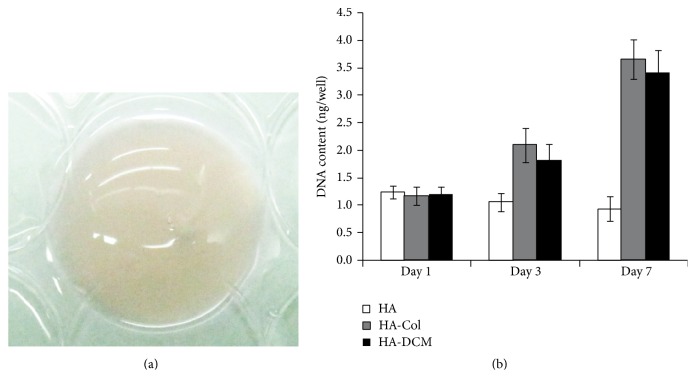
Cell growth of hADSCs in hydrogels. (a) Overall observation of cell-hydrogel constructs indicated that they are transparent. (b) DNA content that was determined by PicoGreen assay indicated that the proliferation of hADSCs in HA-Col and HA-DCM hydrogel was shown to be more than cells in HA-DCM hydrogel significantly.

In order to evaluate the cell growth of hADSCs in the HA-DCM hydrogel, we labeled hADSCs with GFP before embedding in hydrogel. DNA was extracted to determine the proliferation of hADSCs in HA-DCM hydrogel at different time points (Figure 3). On day 1, similar DNA contents of hADSCs in HA, HA-Col, and HA-DCM hydrogel were detected, indicating that no significant deviation was produced when incorporating MSCs into HA, HA-Col, and HA-DCM hydrogel. That is, equal quantities of hADSCs were incorporated into the three hydrogels. On day 3 and day 7, a significant increase of DNA contents was detected in HA-based composite hydrogel, indicating a significant cell growth. However, DNA contents from HA hydrogel maintained a similar level to day 1, suggesting that no evident proliferation of incorporated cells occurred.

### 3.4. Differentiation of hADSCs in Hyaluronic Acid/Acellular Matrix Composite Hydrogel

**Figure 4:**
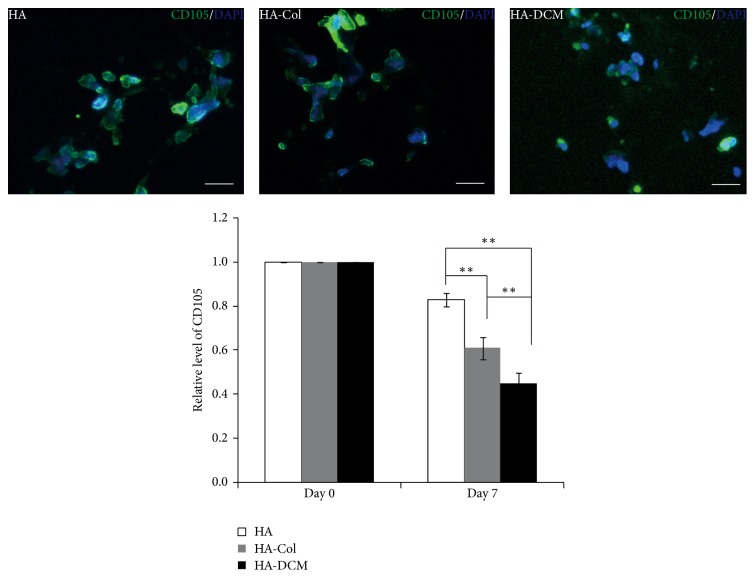
The expression of surface marker CD105 in hADSCs in HA, HA-Col, and HA-DCM hydrogel. The immunofluorescence staining showed CD105 (green) positive cells in HA, HA-Col, and HA-DCM hydrogel. Nuclei were stained by DAPI (blue). Scale bar = 50 *μ*m. The analysis through ImageJ software indicated that cells in HA-DCM hydrogel expressed higher CD105 than cells in HA-Col and HA hydrogel significantly at day 7. *∗∗* indicated *p* < 0.01.

Through immunofluorescence staining of CD105 (endoglin), we could evaluate the differentiation of hADSCs in varied cultural conditions. CD105 was less expressed in cells in HA-DCM hydrogel than in both HA-Col and HA hydrogel (Figure 4). Expression of CD105 in HA-DCM hydrogel was shown to be the lowest, while the CD105 positive cells in HA composite hydrogel were the most expressed in all the three groups.

### 3.5. Hyaluronic Acid/Acellular Matrix Composite Hydrogel Increased the Secretion of Cytokines from hADSCs

**Figure 5:**
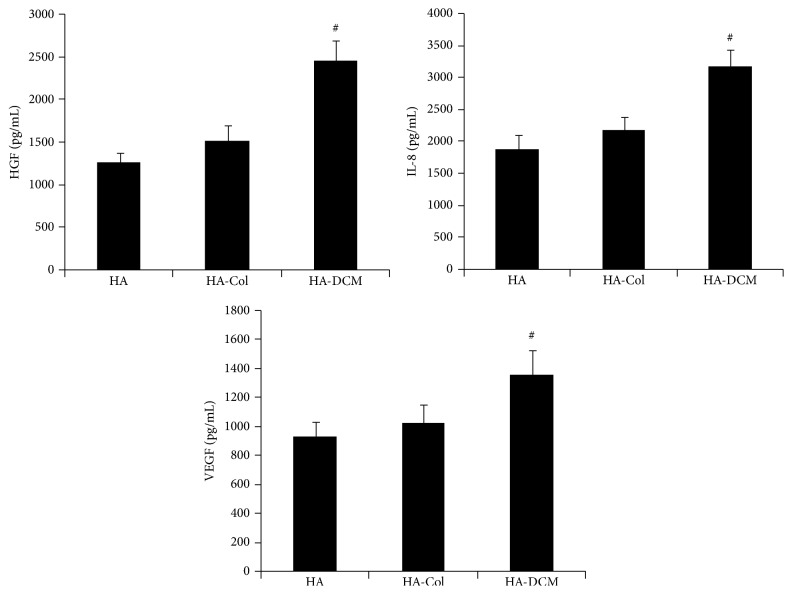
HGF, IL-8, and VEGF release from hADSCs after culture for 3 days in HA, HA-Col, and HA-DCM hydrogel. ELISA assay indicated that hADSCs in HA-DCM composite hydrogel release more HGF, IL-8, and VEGF than cells in HA and HA-Col hydrogel significantly. # indicated *p* < 0.01.

Secretion of cytokines including HGF, IL-8, and VEGF was analyzed by ELISA assay (Figure 5). The concentration of HGF, IL-8, and VEGF in the media was increased significantly by induction of HA-DCM composite hydrogel comparing to HA-Col and HA hydrogel. hADSCs on HA-DCM composite hydrogel secreted HGF (2450 pg/mL) about 1.8 times more than HA group (1340 pg/mL) and HA-Col group (1504 pg/mL). Meanwhile, the concentration of IL-8 in the media of HA-DCM group (3012 pg/mL) was shown to be about 2 times more than in HA group (1843 pg/mL) and HA-Col group (2100 pg/mL). hADSCs in HA-DCM increased secretion of VEGF (1384 pg/mL) significantly compared to HA group (920 pg/mL) and HA-Col group (1015 pg/mL). Therefore, hADSCs in HA hydrogel secreted HGF, IL-8, and VEGF in a similar level to HA-Col group, while HA-DCM composite hydrogel induced hADSCs to secrete more HGF, IL-8, and VEGF significantly than HA and HA-Col group.

### 3.6. Functionalization of hADSCs towards Extracellular Matrix-Producing Vocal Fold Fibroblasts

**Figure 6:**
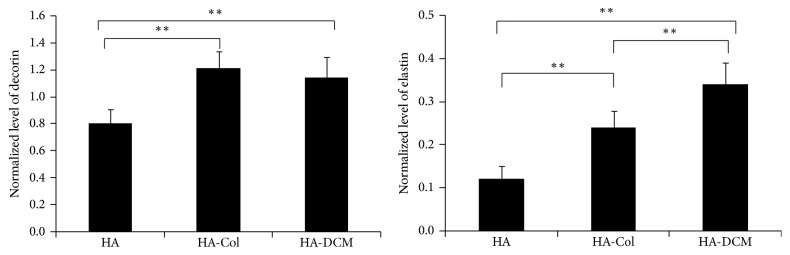
Production of decorin and elastin in hADSCs in HA, HA-Col, and HA-DCM hydrogel. hADSCs in HA-Col and HA-DCM produced more decorin and elastin than cells in HA hydrogel significantly; in particular, the production of elastin was shown to be more than HA-Col and HA hydrogel significantly. *∗∗* indicated *p* < 0.01.

**Figure 7:**
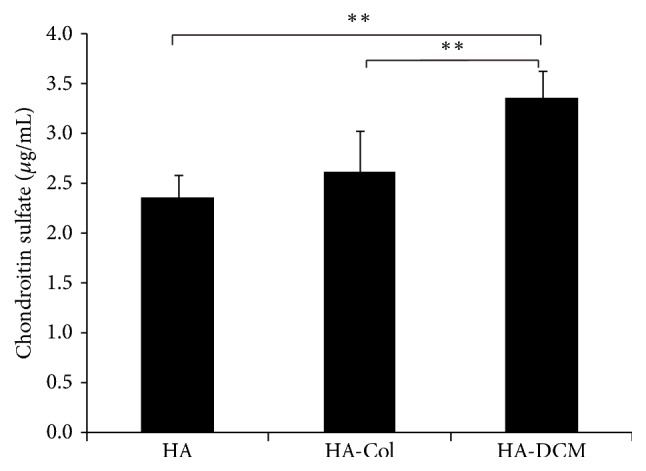
ELISA determined the secretion of chondroitin sulfate of hADSCs in HA, HA-Col, and HA-DCCM hydrogel. The supplementation of DCM in HA hydrogel increased the production of chondroitin sulfate significantly compared to HA-Col and HA hydrogel. *∗∗* indicated *p* < 0.01.

Through qRT-PCR, we further determined the expression of decorin and elastin, which were associated with the vocal fold fibroblasts significantly. The results showed that hADSCs expressed decorin with similar level in HA-Col and HA-DCM hydrogel and increased significantly compared to HA groups. Meanwhile, HA-DCM hydrogel promoted the expression of elastin significantly compared to HA-Col group and HA group. The expression of elastin of hADSCs in HA hydrogel was shown to be the lowest in the three groups (Figure 6). Moreover, the secretion of chondroitin sulfate of hADSCs was promoted by the supplementation of DCM in HA hydrogel significantly compared to hADSCs in HA and HA-Col hydrogel (Figure 7). Therefore, HA-DCM enhanced the differentiation of hADSCs towards vocal fold fibroblasts, which evidenced the notion that HA-DCM hydrogel was promising in vocal fold tissue engineering.

## 4. Discussion

Cell and hydrogel-based regenerative medicine was shown to be outstanding for regeneration of injured tissues or organs. A variety of stem cells have been applied in tissue engineering to construct engineered tissues [27, 28], and so we focused on human adipose tissue derived stem cells because this kind of stem cells is distributed in the abdominal subcutaneous fat tissue abundantly. Meanwhile, the multipotent nature of hADSCs and putative mesenchymal origin of fibroblasts motivated us to explore the fibroblastic differentiation potential of hADSCs for vocal fold regeneration. Lee et al. stated that the injection hADSCs into vocal folds of canine models promoted the repair of tissues significantly [29].

In tissue engineering, scaffolds providing growth environment including necessary growth factors and 3D spatial environment were shown to be critical for growth and differentiation of stem cells [6]. The extracellular environment determined the potentials of cell growth and differentiation, such as the stiffness of substrates [30–33]. In the present study, hADSC proliferated evidently in HA-Col and HA-DCM hydrogels, but it was not observed in HA hydrogel (Figure 4). The main reason should be ascribed to the property of the material itself; that is, HA hydrogel alone may have limited biocompatibility or bioactivity to support the proliferation of incorporated hADSCs. Actually, this has been confirmed by a previous report too [17]. The result suggested that though it is an important ECM constituent in vocal fold lamina propria, hyaluronic acid alone may not be suitable to be used as stem cell scaffold for vocal fold tissue engineering. Modifying HA with other hydrogels, or constructing cogels of HA with other hydrogels as investigated in the present and previous study [17], is highly required.

Previously,* in vitro* studies with application of stem cells and hydrogel in vocal fold tissue engineering showed that cogels of HA-Col or HA-fibrin promoted the proliferation, differentiation, and synthesis of elastin [17]. Our results indicated that the supplementation of DCM promoted cell elongation and differentiation significantly compared to HA-Col and HA. We think that this was probably related to the scaffold environment and some signal molecules that were provided by HA-Col hydrogel. As is known, the differentiation of stem cells was mainly regulated by the extracellular environment, signal molecules, and cell-cell interaction [34]. On the one hand, we supposed that HA-DCM hydrogel may provide a more favourable environment than HA and HA-Col hydrogels for hADSC growth and differentiation. On the other hand, DCM has been shown to be with multiple growth factors and functional proteins for cell growth and differentiation, such as bFGF, TGF-*β*, or BMP4 [35–38]. These proteins may also function as signal molecules stimulating the differentiation of incorporated hADSCs HA-DCM hydrogel. Therefore, hADSCs elongated and differentiated better in HA-DCM hydrogel than in HA and HA-Col hydrogels as observed in the study.

We also found that the supplementation of DCM in HA hydrogel induced the secretion of HGF, IL-8, and VEGF of hADSCs, which might be involved in the promotion of cell growth, elongation, and differentiation by cogel of HA-DCM. Previous studies showed that HGF as an important regulatory factor was secreted by ADSCs* in vitro* significantly to repair the scar fibroblasts of vocal fold [39]. HGF could reduce the deposition of collagen I and increase HA to prevent fibrosis of the vocal fold [40]. The injection of decellularized small intestinal submucosa gel containing HGF repaired and remodeled injured vocal fold* in vivo* significantly [41]. Therefore, the secretion of HGF would increase the therapeutic efficiency of hADSCs significantly.

VEGF as another important paracrine factor was increased by HA-DCM hydrogel, and this suggested that cell-hydrogel constructs may potentially promote ECM remodeling and regulation, angiogenesis, and normal wound-healing process [42]. We observed that the synthesis of important components of ECM such as decorin and elastin has been promoted by cogel of HA-DCM. HGF were confirmed to have a critical effect on the remodeling of injured vocal fold to increase the synthesis of elastin [43].

IL-8 is an important proinflammatory and angiogenic cytokine involved in the regulation of vocal fold remodeling and regeneration. It has been shown that the vocal fold fibroblasts seeding on the surface of Extracel® hydrogels, which resulted from the cross-linking of HA and gelatin, induced proinflammatory cytokines, IL-8 and TNF-*α*,* in vitro* [44]. With the secretion of IL-8, hADSCs in hydrogel can induce the monocytes and macrophages to repair vocal fold injuries by remodeling of ECMs* in vivo* [45]. As for the exact role of IL-8 in the repair of vocal fold, it will be studied in future work.

This study represents our effort in the evaluation of cogel of HA-DCM to foster the fibroblastic differentiation of hADSCs. In our study, cells embedded in cogels of HA-DCM were cultivated under static conditions. hADSCs upregulated the secretion of HGF, IL-8, and VEGF to promote the differentiation of hADSCs in HA-DCM hydrogel.

## 5. Conclusion

Cogel of HA-DCM as a promising scaffold supported the proliferation and prolonged the cell shape of hADSCs significantly compared to HA and HA-Col hydrogel. Moreover, the cogel of HA-DCM promoted the differentiation of hADSCs towards vocal fold fibroblasts through stimulation of secretion of HGF, IL-8, and VEGF and synthesis of extracellular matrix such as elastin and decorin. Collectively, these results demonstrated that HA-DCM hydrogel holds a great potential for vocal fold regenerative applications through the increase of cytokine release and ECM synthesis.
